# Supplementary material for: MetaRibo-Seq measures translation in microbiomes
Source: Nat Commun. 2020 Jun 29;11:3268. doi: 10.1038/s41467-020-17081-z (PMC7324362; doi:10.1038/s41467-020-17081-z)
Supplement: Supplementary file 10 — Supplementary Data 7 [file 41467_2020_17081_MOESM10_ESM.zip › File2/Confidence_VeryHigh_Taxonomy/349586_out.krona.html]

Javascript must be enabled to view this page.

members
magnitude
magnitudeUnassigned
count
unassigned
taxon
rank

349586\_out

7


SRS019068\_contig\_number\_contig-100\_16115.98393
1

superkingdom
6
2

phylum
6
1239

186801
6
class

6
order
186802

541000
family
5

genus

SRS148424\_contig\_number\_contig-100\_329.175099
5
1
1263

2293174
4
species

SRS048164\_contig\_number\_contig-100\_953.182423SRS1041136\_contig\_number\_16750SRS1041145\_contig\_number\_36219SRS893358\_contig\_number\_1157

186803
1
family

1898203
1

SRS012969\_contig\_number\_40859
species
